# Supplementary material for: A low-cost culture- and DNA extraction-free method for the molecular detection of pneumococcal carriage in saliva
Source: Microbiol Spectr. 2024 Jul 19;12(9):e00591-24. doi: 10.1128/spectrum.00591-24 (PMC11370248; doi:10.1128/spectrum.00591-24)
Supplement: Supplemental material — Tables S1-S5; Fig. S1 and S2. [file spectrum.00591-24-s0001.docx]

**Supplementary Information**

**A LOW-COST CULTURE- AND DNA EXTRACTION-FREE METHOD FOR THE MOLECULAR DETECTION OF PNEUMOCOCCAL CARRIAGE IN SALIVA**

Chikondi Peno^1*^, Tzu-Yi Lin^1*^, Maikel Hislop^1^, Devyn Yolda-Carr^1^, Katherine Farjado^1^, Anna York^1^, Virginia Pitzer^1^, Daniel M. Weinberger^1^, Amy Bei^1^, Orchid M. Allicock^1^, Anne L. Wyllie^1^

^1^Department of Epidemiology of Microbial Diseases, Yale School of Public Health, New Haven, Connecticut, USA

*These authors contributed equally

Correspondence:

Anne Wyllie

[anne.wyllie@yale.edu](mailto:anne.wyllie@yale.edu)

60 College St

New Haven

CT 06510

Chikondi Peno

[chikondi.peno@yale.edu](mailto:chikondi.peno@yale.edu)

60 College St

New Haven

CT 06510

**Supplementary Table 1. qPCR C_T_ values for samples yielding non-concordant results for pneumococcal detection following testing using the extraction-free method and the culture-enrichment method.**

| Sample ID | Extraction-free method (C_T_ values) | Culture-enrichment method (C_T_ values) |
| --- | --- | --- |
| Sample 1 | 45 | 33.42 |
| Sample 2 | 45 | 31.73 |
| Sample 3 | 45 | 32.41 |
| Sample 4 | 45 | 36.87 |
| Sample 5 | 45 | 37.12 |
| Sample 6 | 45 | 37.25 |
| Sample 7 | 45 | 38.04 |
| Sample 8 | 45 | 38.43 |
| Sample 9 | 45 | 38.53 |
| Sample 10 | 45 | 38.93 |
| Sample 11 | 45 | 38.97 |
| Sample 12 | 45 | 39.28 |
| Sample 13 | 45 | 39.4 |
| Sample 14 | 45 | 39.49 |
| Sample 15 | 45 | 39.49 |
| Sample 16 | 45 | 39.49 |
| Sample 17 | 45 | 39.59 |
| Sample 18 | 45 | 39.89 |
| Sample 19 | 45 | 39.92 |
| Sample 20 | 45 | 39.94 |
| Sample 21 | 38.85 | 45 |
| Sample 22 | 32.31 | 45 |
| Sample 23 | 34.83 | 45 |
| Sample 24 | 37.1 | 45 |
| Sample 25 | 37.82 | 45 |
| Sample 26 | 38.15 | 45 |
| Sample 27 | 38.39 | 45 |
| Sample 28 | 38.9 | 45 |
| Sample 29 | 39.89 | 45 |

**Supplementary Table 2. Limit of detection for pneumococcus when testing saliva in qPCR**

**following the culture-enrichment and extraction-free sample processing methods.**

|  | **Extraction-free (C_T_ values)** | | | **Culture-enriched (C_T_ values)** | | |
| --- | --- | --- | --- | --- | --- | --- |
| **CFU/mL** | **Replicate1** | **Replicate 2** | **Replicate 3** | **Replicate 1** | **Replicate 2** | **Replicate 3** |
| 5X10^7^ | 18.43 | 18.45 | 18.60 | 12.92 | 12.67 | 12.60 |
| 5X10^6^ | 22.34 | 22.38 | 22.23 | 13.23 | 13.57 | 12.86 |
| 5X10^5^ | 25.07 | 25.33 | 25.27 | 14.31 | 14.62 | 14.92 |
| 5X10^4^ | 28.75 | 29.32 | 29.33 | 18.06 | 17.86 | 17.60 |
| 5X10^3^ | 32.65 | 33.01 | 32.34 | 22.60 | 22.07 | 22.07 |
| 5X10^2^ | 34.05 | 35.55 | 35.46 | 24.59 | 26.06 | 25.03 |
| 5X10^1^ | ND | 37.04 | 36.23 | 34.26 | 30.16 | 29.29 |
| 500 | - | - | - | 24.88 | 26.05 | 24.54 |
| 250 | - | - | - | 26.28 | 26.53 | 26.31 |
| 125 | - | - | - | 26.28 | 26.53 | 26.31 |
| 62.5 | - | - | - | 30.83 | 30.63 | 27.97 |
| 30 | - | - | - | 27.60 | 30.10 | 27.71 |
| 15 | - | - | - | 32.21 | 30.57 | 33.93 |
| 8 | - | - | - | 30.64 | 34.48 | 33.97 |
| 0 (NEG) | ND | ND | ND | ND | ND | ND |

*CFU = colony forming units; ND = not detectable; NEG = negative control*

**Supplementary Table 3. C_T_ value differences obtained using culture-enrichment and extraction-free methods.**

|  | **ΔC_T_ value** | **Standard Error** | ***p*-value*** |
| --- | --- | --- | --- |
| Extraction free method (vs CE method) | +6.69 | 0.311 | <0.00001 |
| School year 2021/2022 | -1.24 | 0.378 | 0.00107 |

**p*-values are derived from a linear regression model

**Supplementary Table 4. C_T_ value relationship between detection method and sampling season.**

|  | **ΔC_T_ value** | **Standard Error** | ***p*-value*** |
| --- | --- | --- | --- |
| Extraction free method (vs CE method) | +5.70 | 0.669 | <0.00001 |
| School year 2021/2022 (vs 2020/2021) | -1.87 | 0.534 | 0.000490 |
| Method: School year interaction | +1.26 | 0.755 | 0.096455 |

**p*-values are derived from a linear regression model

**Supplementary Table 5. Cost for pneumococcal carriage detection in saliva samples using extraction-free protocol or culture-enrichment and DNA extraction.**

| **Items** | **Supplier/Brand** | **Catalog Number** | **Price per sample** | | |
| --- | --- | --- | --- | --- | --- |
|  | | | **Culture-enrichment and DNA extraction** | | **Extraction-free** |
| ***Saliva collection*** |  |  |  |  |  |
| Falcon tubes (50 mL) | Cell Treat | 229435 | $0.37 | | $0.37 |
| Bulb pipette (5 mL) | Fisher Scientific | 13-711-5AM | $0.15 | | $0.15 |
| Cryogenic label | Dymo |  | $0.62 | | $0.62 |
| **Total cost for saliva collection** |  |  | **$1.14** | | **$1.14** |
| ***Saliva culture enrichment*** |  |  | **Commercial gent plate** | **Inhouse gent plate** |  |
| Gent plate | Remel | R01227 | $6.90 |  |  |
| Spreader | Celltreat | 22961 | $0.54 | $0.54 |  |
| Cryovial | Heathrow scientific | HS10060 | $0.28 | $0.28 |  |
| Serological pipette | Fisher Sci | 170355 | $0.08 | $0.08 |  |
| Brain heart Infusion | BD Diagnostics | 237500 | $0.06 | $0.06 |  |
| Glycerol | SigMA-Aldrich | G5516 | $0.04 | $0.04 |  |
| Petri dishes | Research Products International | 160268 |  | $0.25 |  |
| Defibrinated Sheep’s Blood | Colorado Serum Company | 31125 |  | $0.62 |  |
| TSA II | BD Diagnostics | 212305 |  | $0.14 |  |
| Gentamicin (10 mg/mL) | Life technologies | 15710-064 |  | $0.03 |  |
| **Total cost for culture-enrichment** |  |  | **$7.90** | **$2.04** |  |
| ***DNA extraction (MagMAX™ Viral/Pathogen Nucleic Acid Isolation Kit)*** | | | |  |  |
| Magnetic Beads | ThermoFisher | A42362 | $1.25 | |  |
| Proteinase K | ThermoFisher | A42363 | $0.16 | | $0.16 |
| Binding Solution | ThermoFisher | A42359 | $0.67 | |  |
| Wash Buffer | ThermoFisher | A42360 | $0.73 | |  |
| Elution Solution | ThermoFisher | A42364 | $1.00 | |  |
| **Total cost for DNA extraction** |  |  | **$3.81** | | **$0.16** |
| ***Extra items needed when using automated DNA extraction on Apex kingfisher machine*** | | | | | |
| Cryovial tube | Heathrow Scientific | HS10060 | $0.64 | |  |
| Sample/wash plates | ThermoFisher |  | $2.02 | |  |
| Elution plates | ThermoFisher |  | $0.38 | |  |
| Handystep tips (average) | ThermoFisher |  | $2.05 | |  |
| 96 well tip comb for Kingfisher Apex | ThermoFisher |  | $0.29 | |  |
| **Total for DNA extraction** |  |  | **$5.38** | |  |
| ***S. pneumoniae piaB qPCR*** |  |  |  |  |  |
| MMX [SSO Advanced] | BioRad |  | $0.72 | | $0.72 |
| Molecular grade Water | Research Products International |  | $0.01 | | $0.01 |
| Primers | Eurofins |  | $0.01 | | $0.01 |
| Probes | Eurofins |  | $0.07 | | $0.07 |
| PCR plate | BioRad |  | $0.42 | | $0.42 |
| **Total for qPCR detection** |  |  | **$1.23** | | **$1.23** |
| **Total cost per sample** |  |  | **$13.60-$19.45** | | **$2.53** |


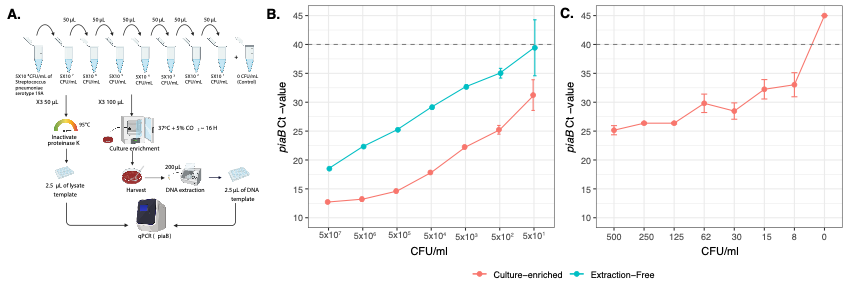
***Supplementary Figure 1.*** ***Limit of detection of pneumococcus using extraction-free and culture-enriched methods.*** *A). Workflow used to evaluate the limit of detection for the extraction-free method. B). Limit of assay detection of the extraction-free saliva compared to culture-enriched saliva. C) Limit of assay detection for culture-enriched saliva. Data shown as mean and standard deviation of biological triplicate. CFU = colony forming units, CE = culture-enriched.*

*
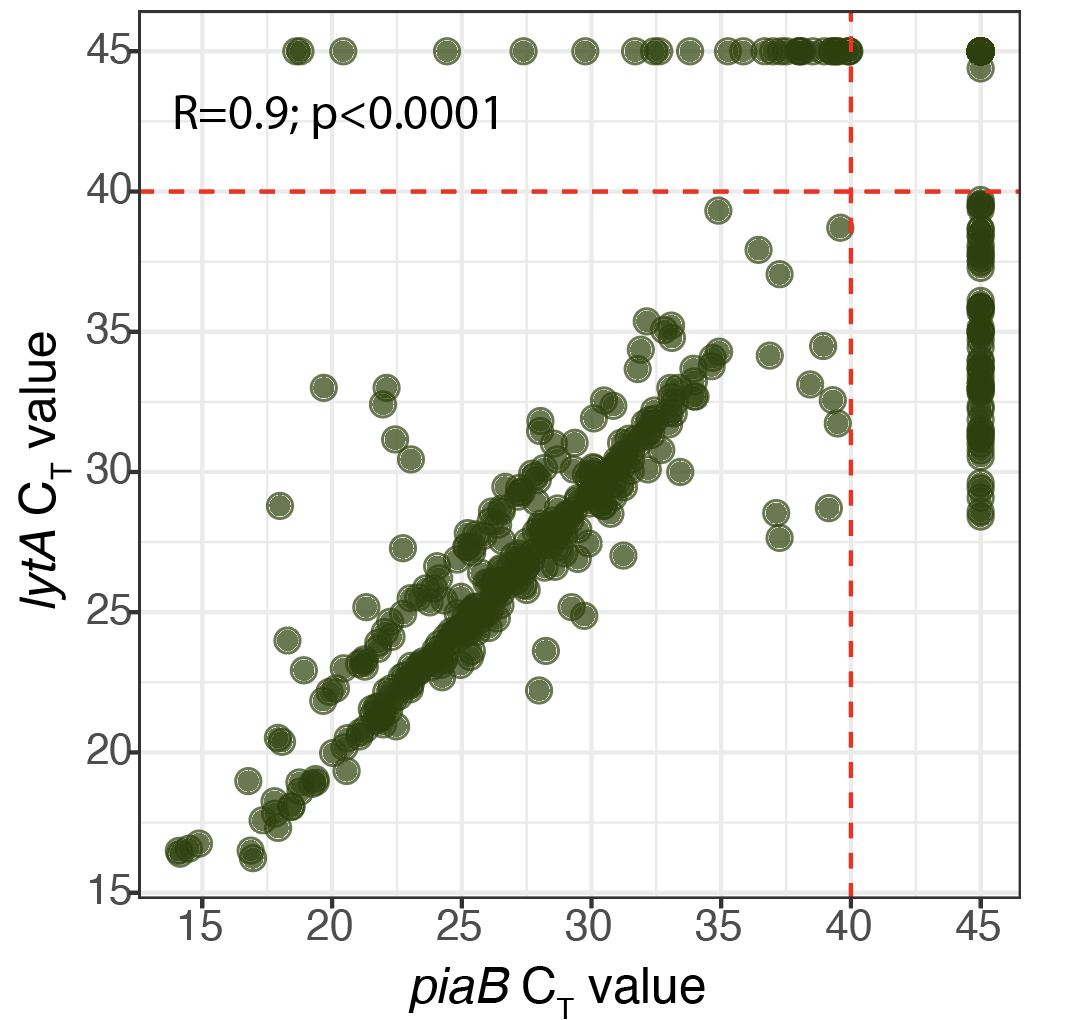
*

***Supplementary Figure 2. Correlation between qPCR*** *C_T_* ***-values obtained when targeting pneumococcal genes, piaB and lytA, when testing DNA templates extracted following culture-enrichment of the saliva samples collected in the study.*** *Scatterplot depicting relationship of C_T_-values for pneumococcus-specific genes, piaB and lytA, obtained following testing of saliva samples processed by the culture-enrichment. The red dotted line marks the threshold assigned to discriminate between positive and negative samples (C_T_-value = 40). Correlation coefficient (R) was obtained using the Pearson correlation test.*
